# Purine metabolism-related gene expression signature predicts survival outcome and indicates immune microenvironment profile of gliomas

**DOI:** 10.3389/fphar.2022.1038272

**Published:** 2022-11-10

**Authors:** Siliang Chen, Shuxin Zhang, Zhihao Wang, Junhong Li, Yunbo Yuan, Tengfei Li, Mingrong Zuo, Wentao Feng, Wenhao Li, Mina Chen, Yanhui Liu

**Affiliations:** ^1^ Department of Neurosurgery, West China Hospital of Sichuan University, Chengdu, Sichuan, China; ^2^ Department of Head and Neck Surgery, School of Medicine, Sichuan Cancer Hospital and Institute, Sichuan Cancer Hospital, University of Electronic Science and Technology of China, Chengdu, China; ^3^ Neuroscience and Metabolism Research, State Key Laboratory of Biotherapy, West China Hospital, Sichuan University, Chengdu, China

**Keywords:** purine metabolism, glioma, tumor microenvironment, prognosis, immune infiltration, immune checkpoint inhibitor

## Abstract

Glioma is the most common malignant tumor in the central nervous system. The impact of metabolism on cancer development and the immune microenvironment landscape has recently gained broad attention. Purines are involved in multiple metabolic pathways. It has been proved that purine metabolism could regulate malignant biological behaviors and response to immune checkpoint inhibitors in multiple cancers. However, the relationship of purine metabolism with clinicopathological features and the immune landscape of glioma remains unclear. In this study, we explored the relationships between the expression of purine metabolism-related genes (PuMGs) and tumor features, including prognosis and microenvironment of glioma, based on analyses of 1,523 tumors from 4 public databases and our cohort. Consensus clustering based on 136 PuMGs classified the glioma patients into two clusters with significantly distinguished prognosis and immune microenvironment landscapes. Increased immune infiltration was associated with more aggressive gliomas. The prognostic Purine Metabolism-Related Genes Risk Signature (PuMRS), based on 11 critical PuMGs, stratified the patients into PuMRS low- and high-risk groups in the training set and was validated by validation sets from multiple cohorts. The high-risk group presented with significantly shorter overall survival, and further survival analysis demonstrated that the PuMRS was an independent prognostic factor in glioma. The nomogram combining PuMRS and other clinicopathological factors showed satisfactory accuracy in predicting glioma patients’ prognosis. Furthermore, analyses of the tumor immune microenvironment suggested that higher PuMRS was correlated with increased immune cell infiltration and gene expression signatures of “hotˮ tumors. Gliomas in the PuMRS high-risk group presented a higher expression level of multiple immune checkpoints, including PD-1 and PD-L1, and a better-predicted therapy response to immune checkpoint inhibitors. In conclusion, our study elucidated the relationship between the expression level of PuMGs and the aggressiveness of gliomas. Our study also endorsed the application of PuMRS to construct a new robust model for the prognosis evaluation of glioma patients. The correlations between the profiles of PuMGs expression and tumor immune microenvironment potentially provided guidance for immunotherapy in glioma.

## Introduction

Glioma is a type of highly aggressive tumor and accounts for approximately 80% of all malignant central nervous system (CNS) tumors (Ostrom et al., 2021). The current standard treatment regime comprises surgery, chemotherapy, and radiotherapy (Stupp et al., 2005). However, even with complete treatment procedures, the prognosis of glioma patients remains unsatisfactory (Weller et al., 2021), especially for glioblastoma, which presents with highly malignant biological features and results in fewer than 20 months of median overall survival (Chinot et al., 2014; Gilbert et al., 2014; Stupp et al., 2015). Therefore, the treatment of glioma urgently needs novel therapy to improve patients’ prognosis.

Immunotherapy, which aims to reduce the immune escape of tumors and enhance anti-tumor immunity delivered by immune cells, has been proven effective in many cancers (Zhang and Zhang, 2020). As a vital compartment of immunotherapy, immune checkpoint inhibitors (ICIs) have succeeded in improving clinical outcomes in many types of cancer, including non-small-cell lung cancer (Reck et al., 2016), melanoma (Larkin et al., 2015), cervical cancer (Tewari et al., 2022), and gastric cancer (Janjigian et al., 2021). However, almost all the phase 3 trials of ICIs failed to improve overall survival in glioblastoma patients (Reardon et al., 2020; Lim et al., 2022; Omuro et al., 2022). CNS’s immunologically quiescent environment is recognized as a potential reason for the failures. Nevertheless, the patients with metastatic brain tumors benefit from ICIs, including metastatic melanoma (Tawbi et al., 2018) and lung cancer (Hendriks et al., 2019), indicating that ICIs can deliver enough anti-tumor capacity to CNS, and the failures in glioma may owe to the distinctive immune microenvironment of glioma. Additionally, neoadjuvant ICIs, including PD-1 inibitors nivolumab and pembrolizumab, could reshape the tumor immune microenvironment and enhance the immune response in glioblastoma (Cloughesy et al., 2019; Schalper et al., 2019), suggesting that the tumor immune microenvironment in glioma could be shifted to be more susceptible to immunotherapy. Therefore, exploring potential pathways to reshape the immune microenvironment and enhance the response to immunotherapy in glioma becomes a focus topic. Furthermore, recent studies demonstrated that interventions targeting the abnormal metabolic features in the tumor might reprogram the immune microenvironment and synergize with ICIs (Li et al., 2019), indicating that targeting the aberrant metabolism of tumors might become a new method to pave the way for immunotherapy.

Purines are critical metabolic precursors for DNA and RNA synthesis in all living cells. Due to the increased growth rate in neoplastic cells (Hanahan and Weinberg, 2011), the demands for purines are enormously upregulated in cancers. Multiple key genes in purine metabolism were also identified as prognostic biomarker in hepatocellular carcinoma (Su et al., 2020). In gliomas, purine synthesis can promote the maintenance of cancer stem cells (Wang et al., 2017). The role of purine metabolism on DNA repair and therapy resistance has also been shown in glioblastomas (Zhou et al., 2020). Besides, purines could also function as the energy currency of cells (ATP and GTP) and signaling molecules (cAMP and cGMP) (Pareek et al., 2021). In addition to direct impact on the anabolic process of tumor cells, purine metabolism could also influence the functional status of the tumor immune microenvironment. Inhibiting purine synthesis in breast cancer was proved to elevate the pyrimidine to purine ratio, increase immunoproteasome expression, and enhance the response to immune checkpoint inhibitors (Keshet et al., 2020). Purinergic receptors on immune cells, including adenosine receptors, inotropic receptors, and metabotropic receptors, played critical roles in regulation of immune response (Cekic and Linden, 2016). For example, macrophages and dendritic cells expressed A2BR, a subtype of adenosine receptor, which was activated by adenosine and functioned to promote macrophages and dendritic cells to release IL-6 and VEGF (Novitskiy et al., 2008; Cekic and Linden, 2016). CD39 and CD73, two essential ectoenzymes in adenosine metabolism, could increase the concentration of anti-inflammatory adenosine and reduce the concentration of pro-inflammatory ATP in microenvironment (Deaglio et al., 2007). The local accumulation adenosine produced by activated CD39 and CD73 has been implicated in immunosuppression progress among patients with AIDS (Nikolova et al., 2011). Inhibition of CD73 also showed synergistic effects with immune checkpoint inhibitors in multiple cancers (Goswami et al., 2020; Turiello et al., 2020; Kim et al., 2021; Tu et al., 2022). These prior findings collectively suggest that purines metabolism was closely associated with malignancy, immune profiles, and responses to immunotherapy in cancers, which inspired us to investigate the purine metabolism in brain gliomas.

In the present study, we utilized multiple cohorts, including TCGA, CGGA, REMBRANDT, and our own patients’ cohort, to explore the relationships between purine metabolism-related gene expression and clinicopathological features of gliomas. Furthermore, we constructed a risk signature system based on purine metabolism-related genes to investigate the potential ability of purine metabolism to predict clinical outcomes in glioma patients. Finally, we also explored and clarified the correlations between purine-metabolism-related genes and the landscape of the immune microenvironment, aiming to provide novel aspects for enhancing response to immunotherapy in glioma.

## Materials and methods

### Patient cohort and data collection

We obtained clinical information and RNA-sequencing data of glioma patients from five public databases (TCGA, CGGA, and REMBRANDT) and our patient cohort. We included those patients with primary oligodendrogliomas, astrocytomas, and glioblastomas into this study. Patients with age < 18 or recurrent gliomas were excluded from this study, because these tumors represent minority of the sample sets with distinct biological features (Louis et al., 2021). The Cancer Genome Atlas (TCGA) cohort consisted of 662 primary gliomas. The fragments per kilobase million (FPKM) and prognosis data of the TCGA cohort were downloaded from the TCGA website (https://portal.gdc.cancer.gov/). FPKM data of 415 primary gliomas in the Chinese Glioma Genome Atlas (CGGA) 693 glioma cohort (which included 415 primary and 278 secondary or recurrent gliomas) and array data of 369 gliomas in the REMBRANDT cohort were downloaded from the CGGA website (http://www.cgga.org.cn/). The genes with too low maximum FPKM values (< 0.1) typically represent sequencing/mapping artifact and were excluded from the analysis (Supplementary Figure S1A,B).

Our cohort consisted of 77 primary glioma patients from West China Hospital. We collected their tumor tissue during resection surgery and sequenced mRNA of these tumor samples. Then the mRNA-sequencing data were quantified using STAR and normalized to FPKM. The overall survival was calculated as the period from surgery to death or the time of the last available interview (censored value). All four cohorts excluded patients younger than 18 years old from the analysis. Detailed information on clinicopathological features is listed in Table 1.

**TABLE 1:** Clinicopathological characteristics of patients in TCGA, CGGA, REMBRANDT, and WCH cohort.

Characteristics	TCGA (*N* = 662)	CGGA (*N* = 415)	REMBRANDT (*N* = 369)	WCH (*N* = 77)
**Age: mean (range)**	46 (18–89)	43 (19–76)	52 (22–87)	46 (19–77)
Gender				
Female	282 (42.6%)	176 (42.4%)	118 (32.0%)	30 (39.0%)
Male	380 (57.4%)	239 (57.6%)	196 (53.1%)	47 (77.0%)
NA	0	0	55 (14.9%)	0
**Histology**				
Astrocytoma	341 (51.5%)	182 (43.9%)	133 (36.0%)	22 (28.6%)
Oligodendroglioma	167 (25.2%)	94 (22.7%)	59 (16.0%)	21 (27.3%)
Glioblastoma	154 (23.3%)	139 (33.5%)	177 (48.0%)	34 (44.2%)
**Grade**				
G2	214 (32.3%)	134 (32.3%)	88 (23.8%)	29 (37.7%)
G3	237 (35.8%)	142 (34.2%)	66 (17.9%)	14 (18.2%)
G4	154 (23.3%)	139 (33.5%)	177 (48.0%)	34 (44.2%)
NA	57 (8.6%)	0	38 (10.3%)	0
**IDH status**				
WT	236 (35.6%)	169 (40.7%)	NA	42 (54.5%)
Mutant	421 (63.6%)	207 (49.9%)	NA	35 (45.5%)
NA	5 (0.8%)	39 (9.4%)	NA	0
**1p/19q codeletion**				
Non-codel	488 (73.7%)	267 (64.3%)	148 (40.1%)	43 (55.8%)
Codel	167 (25.2%)	88 (21.2%)	24 (6.5%)	19 (24.7%)
NA	7 (1.1%)	60 (14.5%)	197 (53.4%)	15 (19.5%)
**TERT promoter status**				
Mutant	340 (51.4%)	NA	NA	23 (29.9%)
WT	156 (23.6%)	NA	NA	30 (39.0%)
NA	166 (25.1%)	NA	NA	24 (31.2%)
**MGMT promoter status**				
Unmethylated	157 (23.7%)	141 (34.0%)	NA	13 (16.9%)
Methylated	472 (71.3%)	195 (47.0%)	NA	35 (45.5%)
NA	33 (5.0%)	79 (19.0%)	NA	29 (37.7%)
**ATRX status**				
Mutant	192 (29.0%)	NA	NA	53 (68.8%)
WT	459 (69.3%)	NA	NA	22 (28.6%)
NA	11 (1.7%)	NA	NA	2 (2.6%)

Abbreviation: TCGA, the cancer genome atlas; CGGA, chinese glioma genome atlas; WCH, west china hospital; IDH, isocitrate dehydrogenase; TERT, telomerase reverse transcriptase; MGMT, O6-methylguanine-DNA, methyltransferase; ATRX, alpha-thalassemia x-linked intellectual disability syndrome; WT, wild type; NA, not available.

### Consensus clustering analysis based on purine-metabolism-related genes

A total of 163 purine metabolism-related genes (PuMGs) were exported from the Molecular Signature Database (MSigDB) with the keyword “purine metabolismˮ and 136 were kept after excluding lowly expressed genes. The detailed list of these PuMGs before and after exclusion was given in Supplementary Table S1. Unsupervised Consensus clustering analysis was performed to elucidate different purine metabolism patterns in gliomas based on the expression level of purine-metabolism-related genes. Specifically, the “ConsensusClusterPlusˮ R package was utilized for the consensus clustering with iterations set to 100 (Wilkerson and Hayes, 2010). The optional cluster number depended on the cumulative distribution function (CDF) curve of consensus index and sample size. Under the premise of a smoothly escalating CDF, we tried to expand the sample size of each cluster. The t-Distributed Stochastic Neighbor Embedding (tSNE) analysis was utilized to visualize the PuMG expression distinctions among all the clusters. Furthermore, we used the PuMG expression and cluster labels in the TCGA cohort to train a random forest model. Based on this model, we subsequently stratified the patients of the other three cohorts.

### Construction and validation of purine-metabolism-related genes risk signature

We constructed a risk signature evaluation system based on the expression of PuMGs to investigate the correlation between PuMG expression and glioma prognosis. Firstly, the TCGA dataset cohort was split into training and test sets with a ratio of 6:4. The other three cohorts were utilized as validation sets. The PuMGs were monitored using the Least Absolute Shrinkage and Selection Operator (LASSO) Cox regression analysis in the training set. The PuMGs whose coefficient was not zero at the lambdas corresponding to maximum C-index in 100 random repetitions of LASSO Cox regression were identified as essential PuMGs in glioma. Then we fitted a final multivariate Cox regression model to the training set with the essential PuMGs and calculated the purine-metabolism-related genes risk signature (PuMRS) using the following formula:

$$PuMRS\;Risk\;Signature=\underset{i=1}{\sum }\left({\beta_{i}*\mathrm{Exp}}_{i}\right)$$ (1)

The *β* and *Exp* represent the coefficients and expression levels of each essential PuMG in the final multivariate Cox regression, respectively. All patients were allocated into PuMRS high-risk, or low-risk groups based on the optimal cut-off value of the PuMRS determined by “surv_cutpointˮ in the R package “survminerˮ with group proportion ≥ 0.3. Moreover, we used the R package “timeROCˮ to illuminate the receiver operating characteristic (ROC) curve in the validation sets of 1, 2, and 3-year survival and calculated the area under the ROC curve (AUC).

### Functional enrichment analysis and immune microenvironment landscape evaluation

Gene set enrichment analysis (GSEA) and over-representation were used to evaluate the differentially expressed genes (DEG) with Gene Ontology (GO) enrichment using the R package “clusterProfilerˮ based on different consensus clusters and different PuMRS risk groups. R package “limmaˮ was utilized to identify differentially expressed genes (DEGs) based on the consensus clusters and PuMRS risk groups. In the process of DEGs identification for GSEA, we stratified the patients into two groups based on consensus clustering result. Those genes with adjusted *p*-value <0.05 and |log_2_FC| > 0.5 were determined as DEGs. The logFPKM matrix was transferred to pathway expression using the R package “GEVAˮ, and the differentially expressed pathways were determined using the package “limmaˮ.

For tumor immune microenvironment landscape evaluation, we utilized the website CIBERSORTx (https://cibersortx.stanford.edu/) to calculate the absolute infiltration fraction of 22 types of immune cells in gliomas based on the LM22 reference gene signature. LM22 is a validated leukocyte gene signature matrix that contains 547 genes distinguishing 22 human hematopoietic cell phenotypes, including seven T-cell types, naïve and memory B cells, plasma cells, natural killer (NK) cells and myeloid subsets. For further details, please refer to the study published by Newman et al. (2015). The immune microenvironment-related scores, including stromal and immune scores, were calculated by the previously reported algorithm, the Estimation of Stromal and Immune cells in Malignant Tumor tissues using Expression data (ESTIMATE) (Yoshihara et al., 2013). In this algorithm, the stromal-related genes were selected from the non-hematopoiesis-related genes that were differentially expressed between tumor cell fraction and match stromal cells fraction separated by laser capture microdissection in multiple cancers. Moreover, we utilized the data previously published by D. Aran et al., which consisted of tumor purity estimated by the ESTIMATE algorithm and the consensus purity estimation (CPE) results, to evaluate tumor purity in gliomas (Aran et al., 2015). To identify immunological phenotypes, we calculated the tumor immunological phenotype (TIP) gene signature to distinguish “coldˮ tumors from “hotˮ tumors using the algorithm described by Wang et al. (2021). Additionally, the TIDE suite (https://tide.dfci.harvard.edu/) was used to deliver *in silico* analysis of T cell exclusion and dysfunction and to predict response to immune checkpoint inhibitors therapy in gliomas.

### Analyses of copy number variation and gene mutation, amplification, and homozygous deletion

We fetched the gene alterations and copy number variations (CNV) data of patients of the TCGA cohort from the cBioPortal database (https://www.cbioportal.org/) to depict the different patterns of gene alterations and CNVs between different consensus clusters and PuMRS risk groups. The R package “maftoolsˮ was utilized to visualize the gene alterations. In addition, the CNV levels were evaluated by the Genomic Identification of Significant Targets in Cancer (GISTIC) score.

### Nomogram construction based on PuMRS and other prognostic factors

Univariate and multivariate Cox regression analyses were utilized to clarify prognosis factors. First, PuMRS and other potential prognostic factors, including tumor grade, age, chemotherapy, radiotherapy, gender, KPS, 1p/19q codeletion, and IDH mutation status, were enrolled in the univariate Cox regression analysis. Furthermore, we enrolled those factors with a *p*-value < 0.05 into multivariate Cox regression analysis to confirm independent prognostic factors. Those factors with a *p*-value < 0.05 in multivariate Cox regression analysis were determined as independent prognostic factors and included in nomogram construction.

The nomograms were constructed with the above independent prognostic factors using the R package “rmsˮ. Furthermore, we utilized the calibration curves to assess the efficacy of nomograms for prognosis prediction in glioma patients.

### Statistical Analysis

The R software (version 3.6.1) was used to conduct all the bioinformatic analyses. The Wilcoxon rank sum test was used to evaluate the differences between two groups for continuous variables, and the Kruskal–Wallis one-way analysis was used on the condition of three or more groups. We used the chi-square test for categorical variables to determine the difference in proportions. For survival analysis, the R package “survminerˮ was used to conduct Kaplan-Meier (K-M) analysis, and the log-rank test tested the differences between K-M curves. Cox regression analysis was performed using the coxph function in the R package “survival,ˮ and the LASSO-Cox regression was conducted using the R package “glmnet.ˮ In liner regression of scatter plots, we used the iterative Grubbs test to remove the outliers, aiming to guarantee the robustness of correlation analyses.

### Ethical approval and consent to participate

Tumor samples and clinical data collection and use were performed strictly with ethics regulations and approved by the institutional review board of West China Hospital (No. 2018.569) based on local ethics regulations and the 1964 Helsinki declaration and its later amendments. In addition, the patients signed written consent for tumor tissue collection and processing.

## Results

### Consensus clustering analysis based on purine-metabolism-related genes unveiled two distinctive glioma subgroups

**FIGURE 1:**
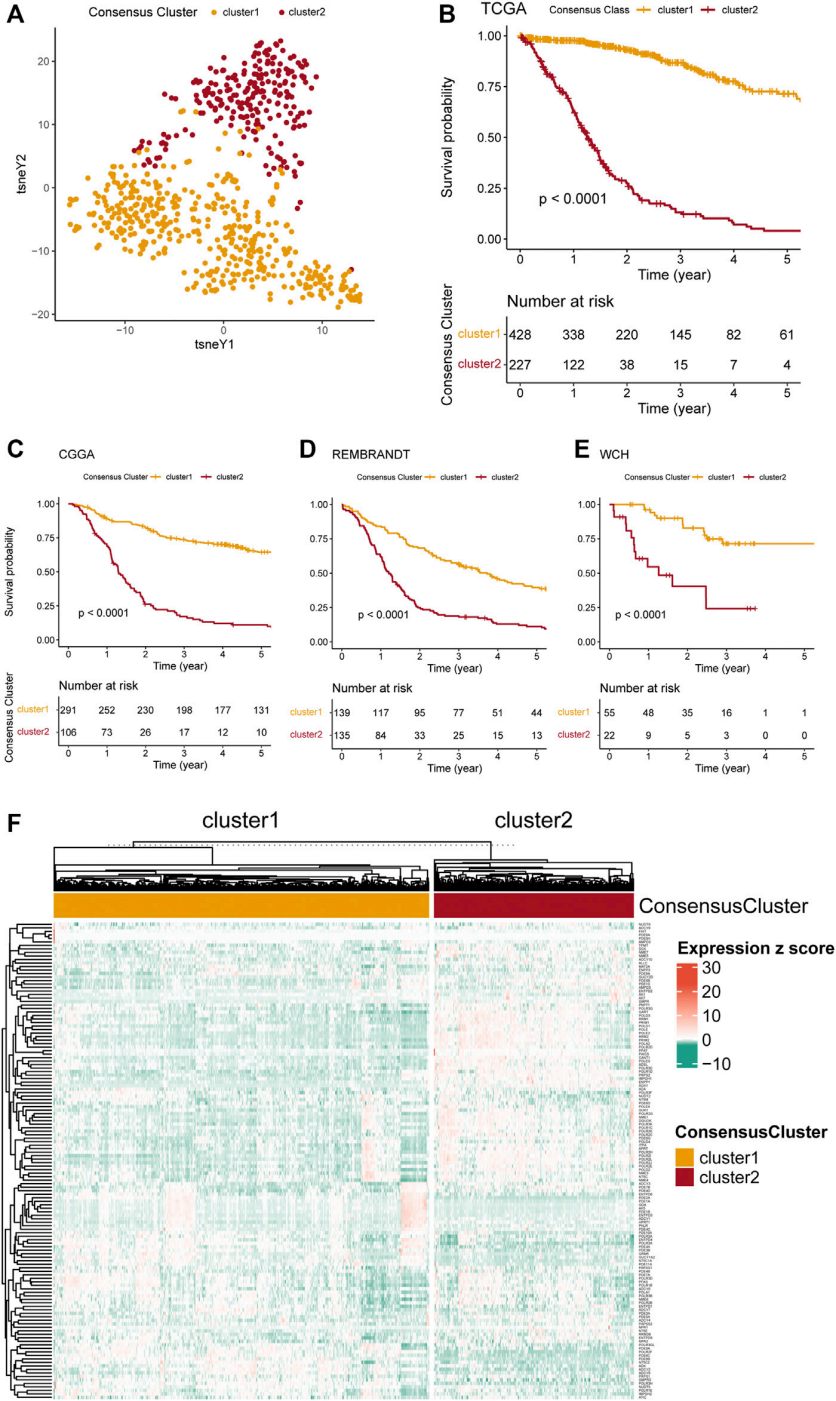
Consensus clustering of gliomas based on PuMGs expression. **(A)** PuMG expression tSNE of the consensus clusters. **(B)** Kaplan-Meier Curve of the consensus clusters in TCGA cohort (*p* < 0.0001). **(C–E)** K-M Curves of the consensus cluster in CGGA, REMBRANDT, and WCH cohorts (*p* < 0.0001). **(F)** Heatmap for the expression levels of 136 PuMGs between the two consensus clusters.

To explore the relationship between PuMGs and gliomas, we performed an unsupervised consensus cluster analysis based on the expression of the 136 PuMGs in the TCGA cohort. After assessing the clusters’ sizes and CDFs based on the principles described in the Materials and Methods section, the gliomas could be classified into two consensus clusters. Their distinction in PuMGs expression patterns was illustrated by tSNE analysis (Figure 1A).

Survival analyses revealed that cluster 1 had enormously better survival outcome than cluster 2 (Figure 1B), with an approximately 60% survival ratio in 5 years. Based on the consensus cluster pattern in the TCGA dataset, we classified the patient of CGGA, REMBRANDT, and WCH cohorts into two clusters to validate the efficacy of clustering. The results revealed that all cluster 1 in these three cohorts showed better prognosis than cluster 2 (Figures 1C–E), indicating that the consensus clustering pattern based on the TCGA dataset promised high efficacy and could be expanded to those cohorts from other datasets. Furthermore, a heatmap was provided to illustrate the expression levels of all 136 PuMGs between two clusters (Figure 1F). The differences in the expression level of all 136 PuMGs were given in Supplementary Figure S1C.

Analyses of clinicopathological features between the two clusters demonstrated noticeable distinctions (Supplementary Figure S2). For example, cluster 2, which showed poorer prognosis, had significantly higher age at diagnosis, higher tumor grade, a higher proportion of MGMT promoter methylation and TERT promoter mutation, and a higher proportion of glioblastoma compared to cluster 1. In addition, more isocitrate dehydrogenase (IDH) mutants, more 1p/19q codeletion, and more alpha-thalassemia x-linked intellectual disability syndrome (ATRX) gene mutation was observed in cluster 1.

**FIGURE 2:**
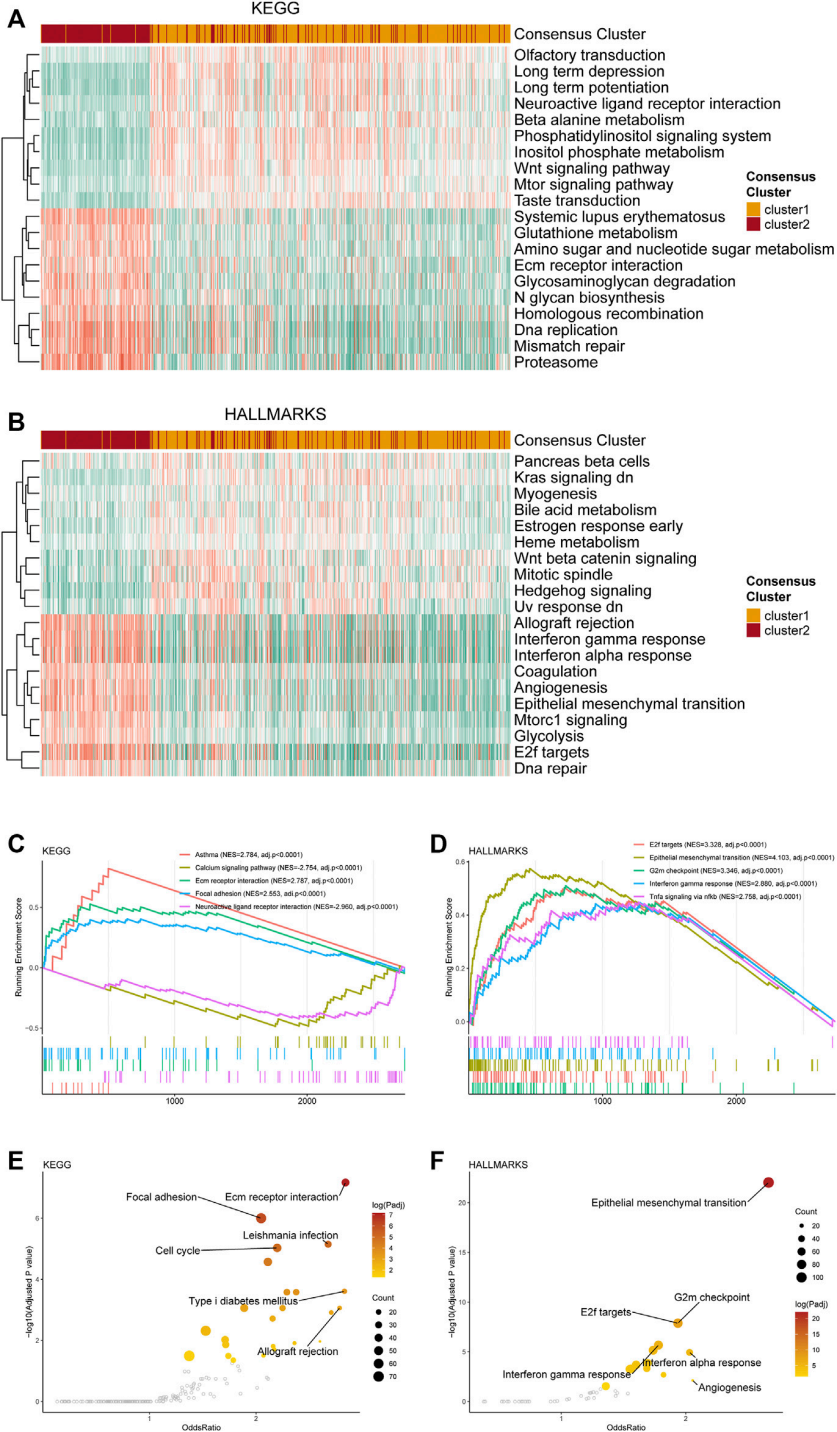
Functional enrichment analysis of the transcriptome of the consensus clusters. **(A)** Top 20 differentially expressed KEGG gene sets. **(B)** Top 20 differentially expressed HALLMARKS gene sets. **(C)** The top five pathways with the highest normalized enrichment score in the KEGG gene sets between two clusters. **(D)** The top five pathways with the highest normalized enrichment score in the HALLMARKS gene sets between two clusters. **(E)** Pathways with high odds ratio and confidence in the KEGG gene sets. **(F)** Pathways with high odds ratio and confidence in the HALLMARKS gene sets.

Distinctive pathway alterations were illustrated in the functional enrichment analysis. For example, the DNA replication pathway, which is tightly related to purine metabolism, was upregulated in cluster 2 (Figure 2A), suggesting more active purine synthesis of cluster 2 to meet the demands of DNA replication. The angiogenesis pathway was also observed to be upregulated in cluster 2 (Figure 2B). Furthermore, the interferon-γ response [normalized enrichment score (NES) = 2.879, adjusted *p*-value < 0.001] and the G2m checkpoint (NES = 3.345, adjusted *p*-value < 0.001) were ranked in the top five gene sets of the HALLMARKS gene sets in the comparison between cluster 1 and 2 (Figure 2D). Besides, the top 5 enriched gene sets in the DEGs between clusters 1 and 2 in the KEGG dataset were also listed (Figure 2C). The detailed NES, *p*-value, and gene list of these pathways were provided in Supplementary Table S2. The normal functional enrichment analysis illustrated the pathway alterations with high odds ratio and high confidence in the KEGG datasets, including extracellular matrix (ECM) receptor interaction, focal adhesion, and cell cycle (Figure 2E). Differences in the epithelial-mesenchymal transition and E2F targets of the HALLMARKS dataset were demonstrated with high odds ratios and confidence (Figure 2F).

### Gene alterations and copy number variations analysis between the two PuMG clusters

**FIGURE 3:**
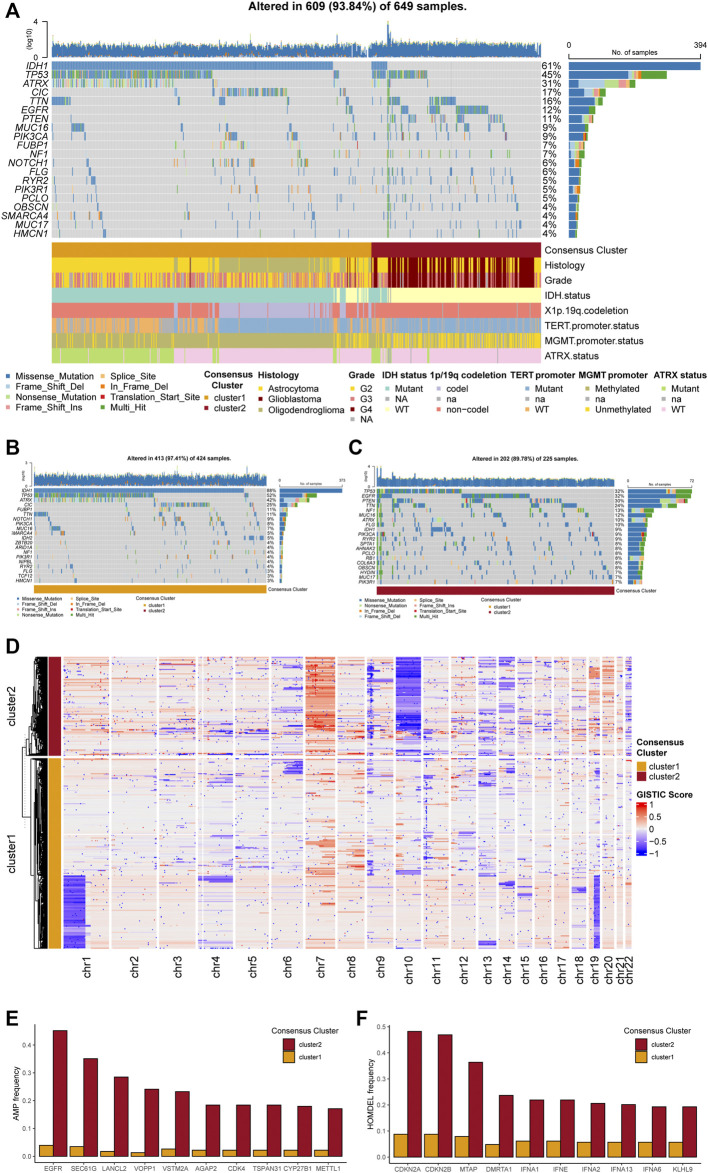
Genetic mutations and copy number variations of the two clusters. **(A)** Gene alterations of top 20 mutated genes. **(B)** Top 20 mutated genes in cluster 1. **(C)** Top 20 mutated genes in cluster 2. **(D)** Heatmap of copy number variations of the two clusters. **(E)** Top 10 amplification genes in two clusters. **(F)** Top 10 homozygously deleted genes in two clusters.

Exploring potential differences in gene patterns between the two clusters, we conducted analyses of gene mutation, gene amplification, gene homozygous deletion, and copy number variations (CNVs). The gene mutation analyses manifested that IDH1, TP53, ATRX, CIC, EGFR, PTEN, and MUC16 were most frequently mutated in the TCGA cohort (Figure 3A). As for each cluster, different gene mutation pattern was depicted. For example, in cluster 1, most glioma samples harbored IDH1 mutation, which was included in the tricarboxylic acid cycle and interreacted with purine metabolism. Besides, TP53, ATRX, and CIC were the other most frequently mutated genes in cluster 1 (Figure 3B). In cluster 2, TP53, EGFR, PTEN, TTN, and NF1 were the top five frequently mutated genes (Figure 3C). The detailed differences in mutations between the two clusters with statistical test results were given in Supplementary Table S3.

The analyses of CNVs depicted different karyotype landscapes between the two clusters (Figure 3D). Gain of chromosome 7 and loss of chromosome 10 (+7/−10), which was determined as a diagnostic marker for glioblastoma linked with poor prognosis, was more frequently observed in cluster 2, agreeing with clinicopathological features. Several PuMGs located in chromosome 7 or 10, including NUDT5, POLR3A, and PDE1C, were likely to be influenced by +7/−10 (Supplementary Table S1). Besides, 1p/19q codeletion, defined as the specific diagnostic marker for oligodendroglioma, mainly occurred in cluster 1, proving that most oligodendrogliomas were located in cluster 1. In the analysis of gene amplification and homozygous deletion, we found that the frequency of EGFR gene amplification was enormously higher in cluster 2 (Figure 3E). The SEC61G, LANCL2, and VOPP1, located in the same locus as EGFR, were also observed with high amplification frequency in cluster 2. Moreover, the homozygous deletion of CDKN2A/B, which was recognized as an indicator for more malignant biological behaviors in gliomas, was significantly more frequently observed in cluster 2 (Figure 3F), in line with the survival analysis.

### Differential analyses of immune features in tumor microenvironment between two clusters

**FIGURE 4:**
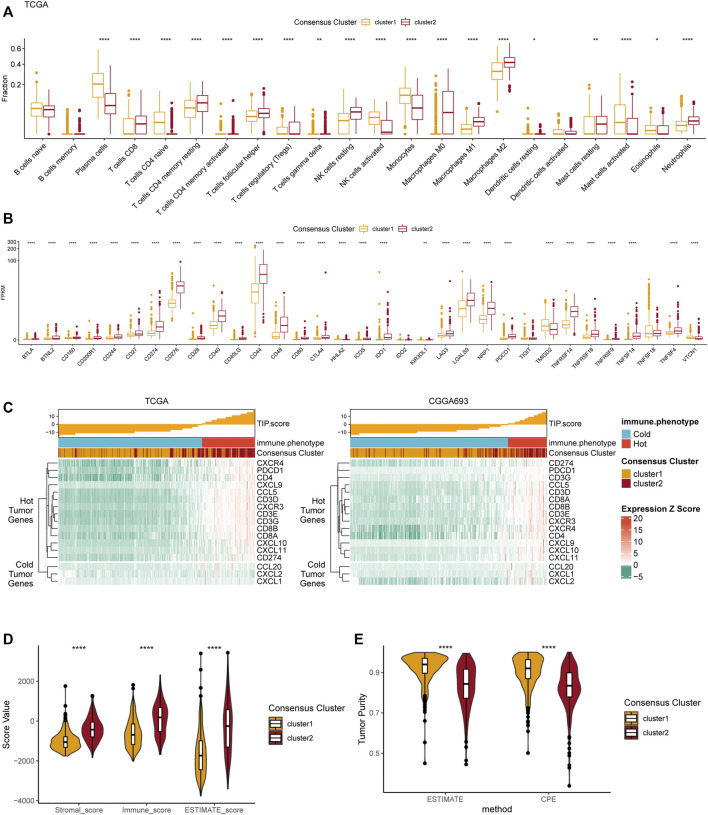
Differences in immune features of tumor microenvironment between two clusters. **(A)** Boxplot of the estimated fraction of 22 immune cells in tumors. **(B)** The expression level of 33 immunotherapy-related genes in each consensus cluster. **(C)** TIP scores and related gene expression levels between two clusters in TCGA and CGGA datasets. **(D)** Stromal, immune, and ESTIMATE scores of the consensus clusters. **(E)** Tumor purity is calculated by the ESTIMATE and CPE algorithms. *p < 0.05; **p < 0.01; ***p < 0.001; ****p < 0.0001.

We performed several analyses based on the two consensus clusters to explore the relationship between purine metabolism and the immune microenvironment in glioma. First, the infiltration fraction of 22 immune cells in the tumor microenvironment (TME) was evaluated using the CIBERSORTx algorithm. Results manifested that more macrophages (M0, M1, and M2), resting NK cells, CD8^+^ T cells, and neutrophils infiltrated into the TME in cluster 2 (Figure 4A). On the contrary, plasma cells infiltration of cluster 1 was significantly more than cluster 2. Furthermore, we conducted a differential analysis based on two clusters to investigate the expression level of several immune-related genes in TME. The results demonstrated that CD274 (PD-L1), CD276 (B7-H3), CD44, and PDCD1 were significantly highly expressed in cluster 2, indicating a more complex immune microenvironment than cluster 1.

The TIP score, calculated according to the expression 12 hot tumor–related genes and 3 cold tumor–related genes, was used to identify the differences in immune phenotype which was shown to be associated with response of tumors to immunotherapy. In line with the above results, cluster 2 showed with higher TIP scores, suggesting that gliomas in cluster 2 were more likely to be immunologically “hotˮ tumors compared to those in cluster 1 (Figure 3C). This phenomenon was also validated in the CGGA dataset. Additionally, the ESTIMATE results revealed that the stromal score, immune score, and ESTIMATE score were significantly higher in cluster 2 compared to cluster 1 (Figure 4D). Finally, the results of tumor purity analysis indicated that the tumor purity in cluster 1 was significantly higher than in cluster 2, suggesting a purer tumor microenvironment in cluster 1 (Figure 4E).

### Construction and validation of purine-metabolism-related genes risk signature and correlation with clinicopathological features

**FIGURE 5:**
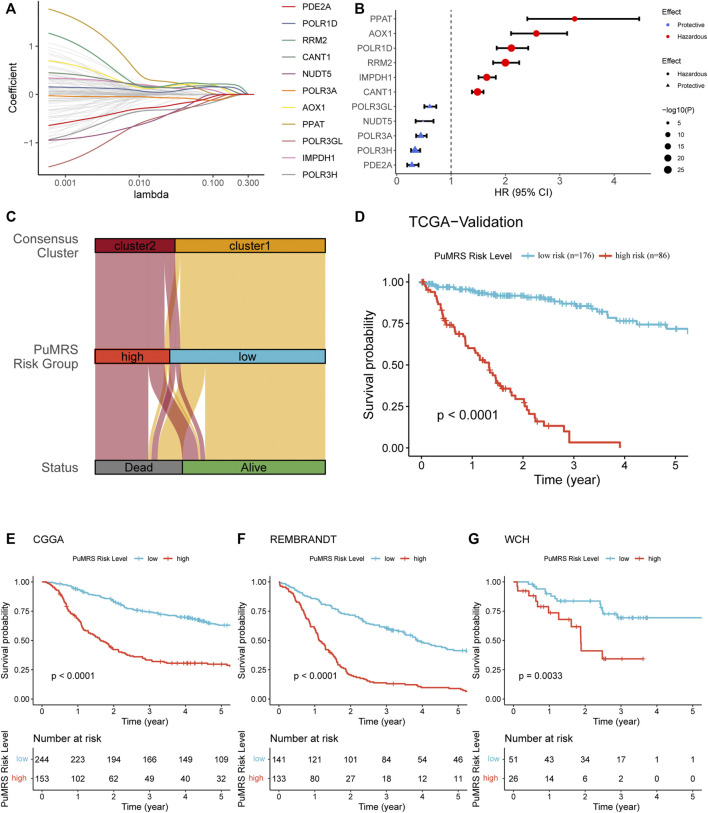
Expression signature of PuMGs and its relationship with prognosis. **(A)** Average of coefficients of 11 critical PuMGs in the LASSO Coxregression at each lambda value. **(B)** The effect of every critical PuMG on the prognosis of glioma. **(C)** The relationship between the consensus clusters and two CRGRS risk groups. **(D)** K-M curve of the TCGA validation set, cut off = −1.566. **(E)** K-M curve of the CGGA cohort, cut off = −3.433. **(F)** K-M curve of the REMBRANDT cohort, cut off = 1.128. **(G)** K-M curve of the WCH cohort, cut off = −0.113.

To identify essential genes for PuMRS construction, we filter 136 purine metabolism-related genes using the LASSO Cox regression with the training set data. Finally, 11 purine-metabolism-related genes, including PDE2A, POLR1D, RRM2, CANT1, NUDT5, POLR3A, AOX1, PPAT, POLR3GL, IMPDH1, and POLR3H, were determined as essential genes for PuMRS construction (Figure 5A). The PuMRS was calculated using the following formula:

0.270*AOX1+0.080*PPAT+0.075*POLR1D+0.048*CANT1+0.038*RRM2+0.030*IMPDH1−0.040*PDE2A−0.058*POLR3H−0.062*POLR3A−0.071*POLR3GL−0.108*NUDT5 (2)

Among these 11 genes, POLR3A, POLR3H, POLR3GL, NUDT5, and PDE2A were determined as protective factors for glioma patients (Figure 5B). The other seven genes were determined as hazardous factors. To validate the prognostic effects of these genes, we utilized the representative immunohistochemical (IHC) staining for POLR1D and PDE2A from the Human Protein Atlas (Pontén et al., 2008) (https://www.proteinatlas.org/). These IHC staining revealed that the protein expression level of POLR1D was higher in high-grade glioma than low-grade glioma (Supplementary Figure S3A), and the protein expression level of PDE2A was lower in high-grade glioma (Supplementary Figure S3B), in accordance with the results from bioinformatic analyses that POLR1D was a hazardous and PDE2A was a protective factor. Then we explored the optimal PuMRS cut-off using the “surv_cutpointˮ algorithm and allocated the patients into PuMRS low- and high-risk groups. Compared with the consensus clustering, we found that most patients of cluster 1 were allocated to the low-risk group, and most cluster 2 patients were allocated to the high-risk group (Figure 5C). The survival analyses confirmed that the glioma patients in the high-risk group had significantly poorer overall survival than the low-risk group in all four validation cohorts (Figures 5D–G).

**FIGURE 6:**
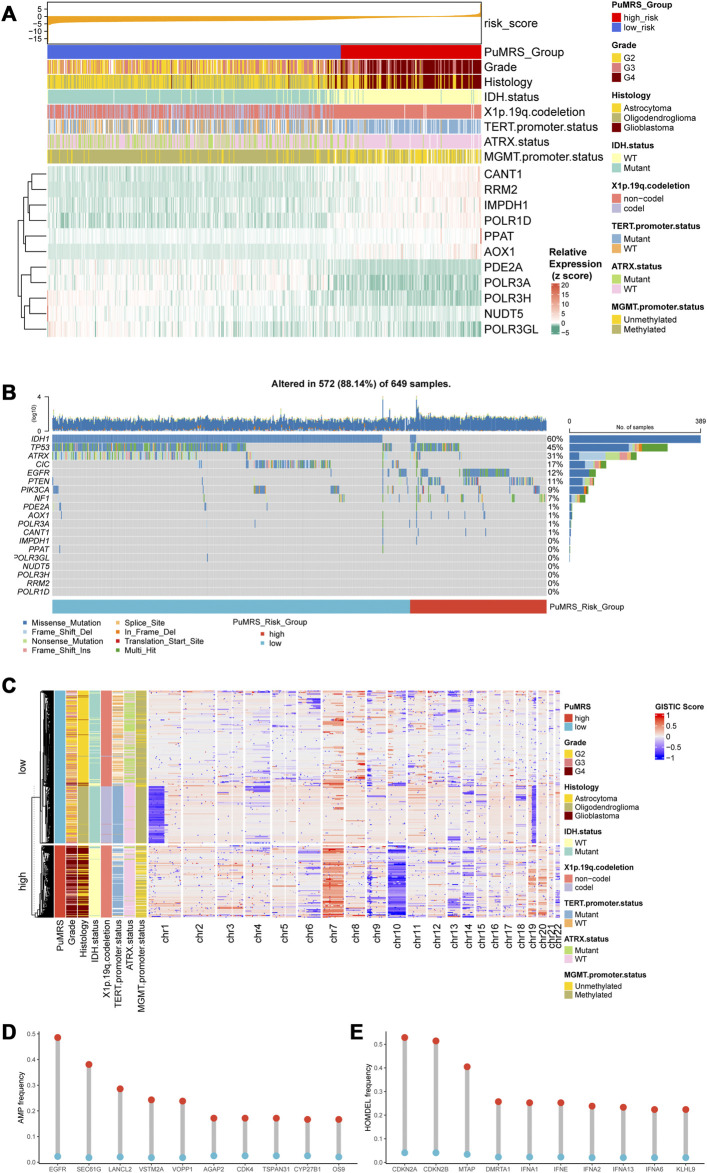
Genetic mutations and copy number variations of the two risk groups. **(A)** Expression level of 11 critical PuMGs and clinicopathological features. **(B)** Gene mutations of 11 critical PuMGs and top 8 frequently mutated genes. **(C)** Heatmap of copy number variations of the two risk groups. **(D)** Top 10 amplification genes in two risk groups. **(E)** Top 10 homozygously deleted genes in two risk groups.

The analyses of clinicopathological features according to the PuMRS risk groups depicted the differences between these two groups. The PuMRS high-risk group significantly had higher tumor grade, more TERT promoter mutation, less IDH mutation, less 1p/19q codeletion, less MGMT promoter methylation, and less ATRX mutation (Figure 6A). Gene mutations analysis demonstrated that the mutation frequency of the 11 essential PuMRS genes was shallow, which contributed to excluding the potential bias effects caused by a gene mutation (Figure 6B). The detailed differences in mutations between the two PuMRS risk groups with statistical test results were given in Supplementary Table S4. Furthermore, the CNVs analysis demonstrated similar patterns with consensus clustering (Figure 6C). The amplification frequency of EGFR, SEC61G, and LANCL2 was significantly higher in the high-risk group compared to the low-risk group (Figure 6D). In addition, the homozygous deletion of CDKN2A/B was frequently observed in the high-risk group (Figure 6E). Detailed analyses of differences in clinicopathological features between the two risk groups were given in Supplementary Figure S4.

### Prediction of glioma prognosis with PuMRS-Based nomograms

**FIGURE 7:**
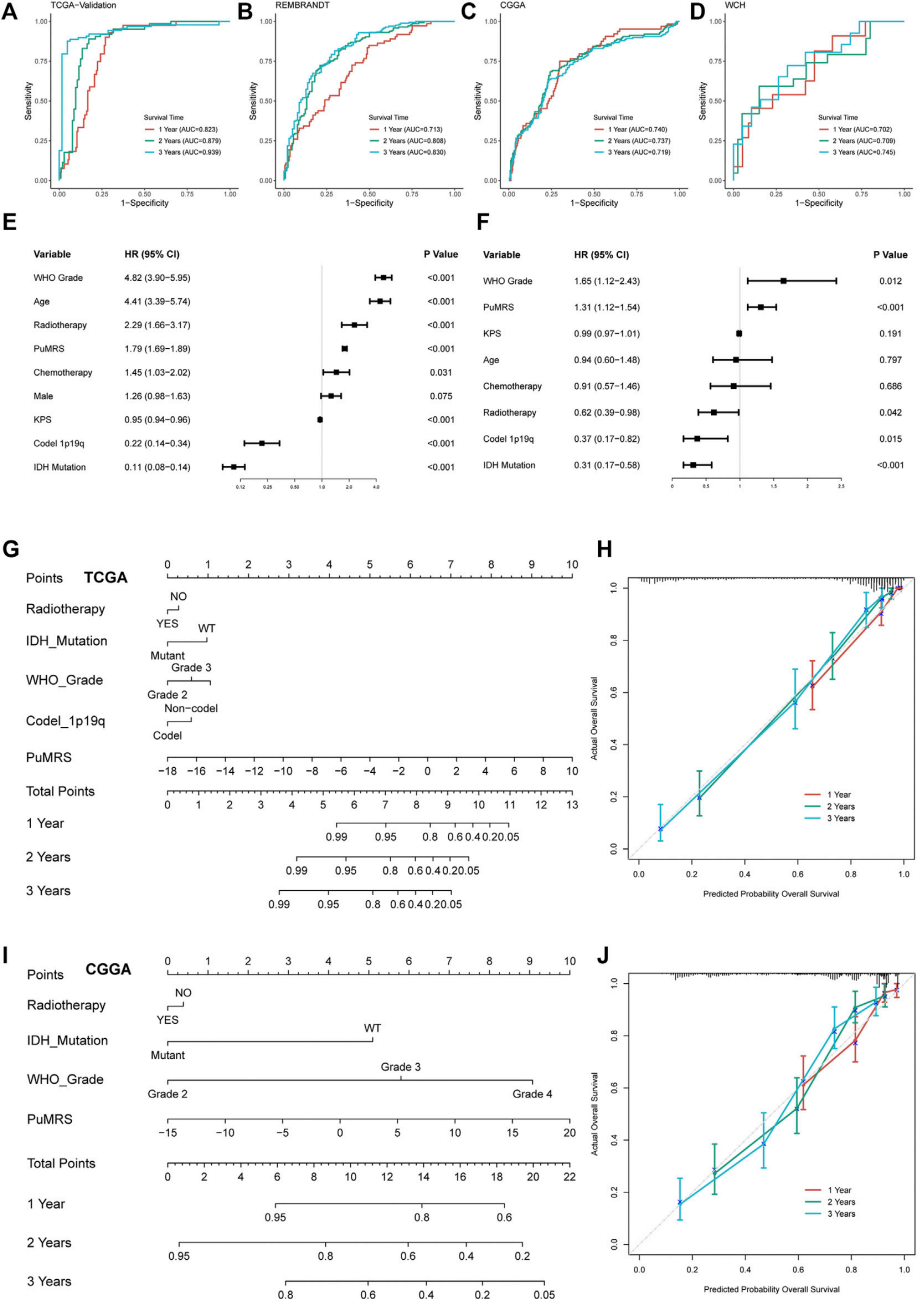
Prognostic value of PuMRS and construction of PuMRS-based nomograms. ROC curves and matched AUC of 1-, 2-, and 3-year survival in **(A)**TCGA validation set, **(B)** CGGA cohort, **(C)** REMBRANDT cohort, and **(D)** WCH cohort. **(E)** Univariate and **(F)** Multivariate Cox regression analysis of potential prognostic factors in overall survival of glioma. Nomogram of 1-, 2-, and 3-year survival of glioma patients based on **(G)** TCGA cohort, **(I)** CGGA cohort. Calibration plots of the nomogram based on **(H)** TCGA cohort and **(J)** CGGA cohort.

To predict the efficiency of PuMRS in predicting glioma prognosis, we first conducted ROC analyses to evaluate the performance of PuMRS alone in predicting glioma patient survival at 1, 2, and 3 years. In the TCGA validation cohort, the AUCs of PuMRS at 1, 2, and 3 years were 0.823, 0.879, and 0.939, respectively (Figure 7A). Similar performances were achieved in the other three validation cohorts (Figures 7B–D). Furthermore, the univariate cox regression analysis demonstrated that PuMRS, together with other factors, including tumor grade, age, radiotherapy, KPS, 1p/19q codeletion, and IDH mutation (*p*-value < 0.05), was a potential prognostic factor in glioma patients (Figure 7E). Subsequently, these factors were enrolled in multivariate Cox regression analysis to verify independent prognostic factors. The result of multivariate Cox regression analysis showed that PuMRS was an independent prognostic factor (*p*-value < 0.05), along with tumor grade, radiotherapy, 1p/19q codeletion, and IDH mutation (Figure 7F).

The nomogram construction of the TCGA cohort was based on these five independent prognostic factors (Figure 7G). The corrected C-index of this integrated nomogram was 0.857, and the calibration curves of 1-, 2-, and 3-year endorsed the accuracy of the nomogram (Figure 7H). Under the same process, the PuMRS was also confirmed as an independent prognostic factor in the CGGA cohort, and a nomogram of the CGGA cohort was based on the PuMRS, along with radiotherapy, IDH mutation, and tumor grade (Figure 7I). Again, the calibration curves also endorsed the accuracy (Figure 7J).

### Analyses of immune characteristics of tumor microenvironment based on PuMRS risk groups

**FIGURE 8:**
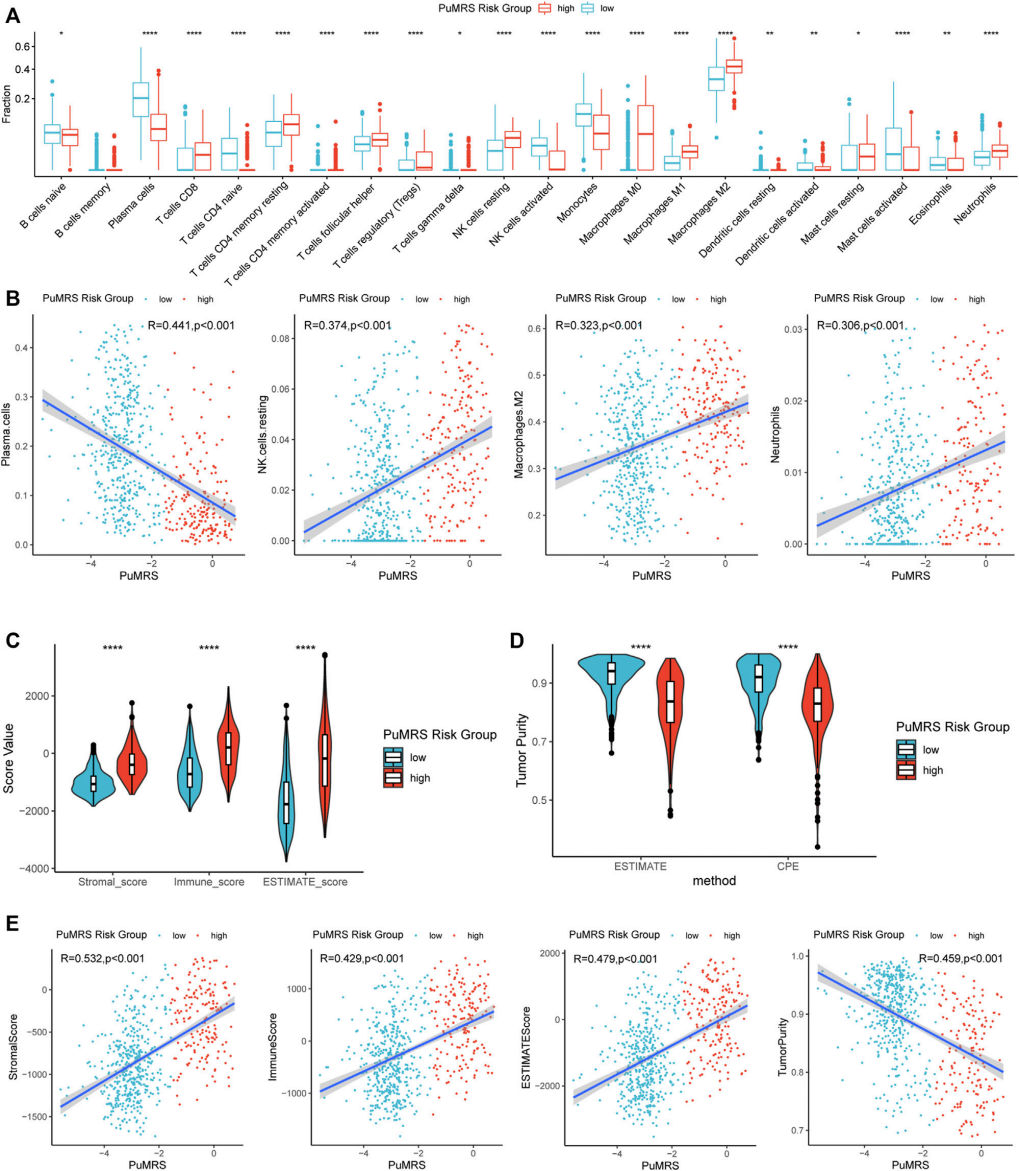
Differences in immune features of tumor microenvironment between two PuMRS risk groups. **(A)** Boxplot of the estimated fraction of 22 immune cells in tumors. **(B)** Analyses of correlations of PuMRS with the infiltration of plasma cells, resting NK cells, M2 macrophages, and neutrophils. **(C)** Stromal, immune, and ESTIMATE scores of the two risk groups. **(D)** Tumor purity of the two risk groups based on the ESTIMATE and CPE algorithms. **(E)** Analyses of correlations of PuMRS with the stromal, immune, ESTIMATE score, and tumor purity. *p < 0.05; **p < 0.01; ***p < 0.001; ****p < 0.0001.

The differences in immune cell infiltration were evaluated using the CIBERSORTx algorithm. The infiltration fractions of CD8^+^ T cells, regulatory T cells (Tregs), resting NK cells, macrophages (M0, M1, M2), and neutrophils were significantly lower in the low-risk group compared to the high-risk group (Figure 8A) in the TCGA cohort. On the contrary, the infiltrations of activated NK cells, plasma cells, and monocytes were higher in the PuMRS low-risk group. Furthermore, the correlation analyses demonstrated that the infiltration of resting NK cells, M2 macrophage, and neutrophils were positively correlated with the PuMRS score (Figure 8B), and the infiltration of plasma cells was negatively correlated with the PuMRS score, indicating that PuMRS could become a prediction tool for tumor immune cell infiltration.

**FIGURE 9:**
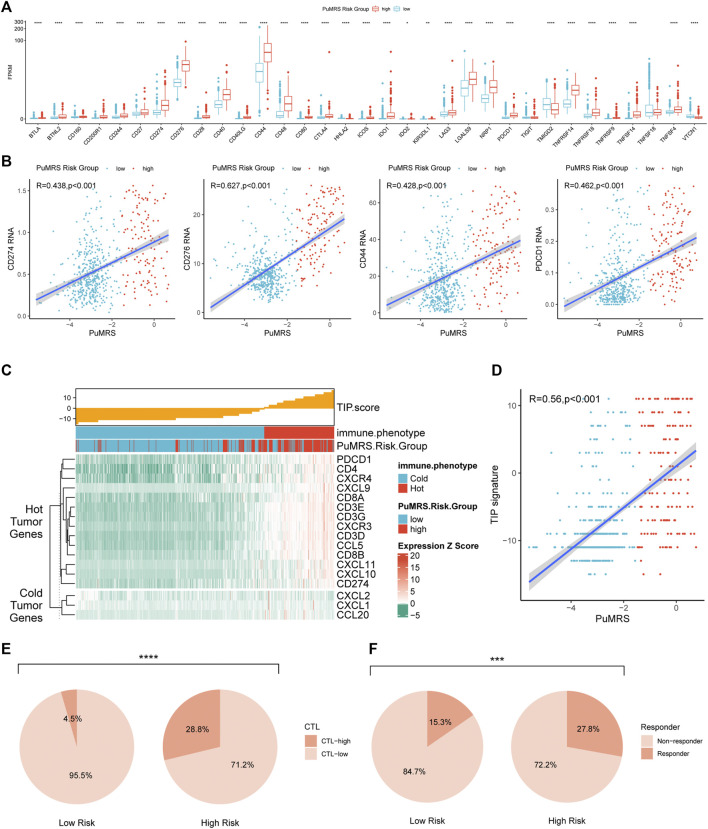
Differences in expression of immunotherapy targets and response to ICIs between two PuMRS risk groups. **(A)** The expression level of 33 immunotherapy-related genes in two risk groups. **(B)** Analyses of correlations of PuMRS with the expression of CD274, CD276, CD44, and PDCD1. **(C)** TIP scores and related gene expression levels between two risk groups in TCGA datasets. **(D)** Analyses of correlations of PuMRS with the TIP score. **(E)** Percentage of predicted CTL level in each risk group. **(F)** Percentage of predicted responders to immune checkpoint inhibitors therapy in each risk group.

Furthermore, the ESTIMATE algorithm was utilized to analyze the immune scores of each PuMRS risk group. Results depicted that the stromal, immune, and ESTIMATE scores were remarkably higher in the PuMRS high-risk group (Figure 8C). Furthermore, the tumor purity of the PuMRS high-risk group was significantly lower than the low-risk group (Figure 8D), suggesting that tumors of the high-risk group might have more immune infiltration and a more complex tumor microenvironment. The correlation analyses revealed that the stomal score, immune score, and ESTIMATE score were positively correlated with PuMRS, and the tumor purity was negatively correlated with the PuMRS (Figure 8E), indicating the potential ability of PuMRS to predict immune-related scores. Additionally, analyses of the expression of immunotherapy-related genes demonstrated that CD274 (PD-L1), CD276 (B7-H3), CD44, and CD279 (PDCD1) were overexpressed in PuMRS high-risk group compared to the low-risk group (Figure 9A). The correlation analysis demonstrated that the expression level of these four immunotherapy-related genes was positively correlated with the PuMRS (Figure 9B), endorsing the ability of PuMRS to predict the expression of immunotherapy-related genes. Immune phenotype analysis revealed that most tumors in PuMRS high-risk group were immunologically “hotterˮ than those in the low-risk group (Figure 9C). The correlation analysis supported this conclusion, which manifested a positive correlation between the TIP score and PuMRS (Figure 9D). Moreover, a higher proportion of cytotoxic T lymphocytes (CTL) was confirmed in the PuMRS high-risk group (Figure 9E). Prediction of immunotherapy response using TIDE revealed that patients with high-risk gliomas were more likely to benefit from ICIs (Figure 9F). Most previous findings could be validated in the other three cohorts (Supplementary Figure S5).

## Discussion

According to the cancer statistics of 2020, there was approximately annually 251 thousand death caused by CNS malignant tumors worldwide (Siegel et al., 2021). Glioma accounted for more than 80% of all these cases (Ostrom et al., 2021). Despite researchers from all over the world constantly trying to improve the treatment outcomes for glioma, the overall survival of glioma patients remains unsatisfactory. For example, even with standard treatment, including surgery, chemotherapy, and radiotherapy, the median overall survival of glioblastoma patients, which accounts for over 50% of newly diagnosed brain gliomas, was 14.6 months (Stupp et al., 2005; Stupp et al., 2017). Therefore, researchers have been persistent efforts on multiple novel therapies for glioma. Immunotherapy, targeting the defense effects of the immune system to attack tumor cells, has made breakthroughs in multiple cancers (Eggermont et al., 2018; Gandhi et al., 2018; Choueiri et al., 2021; Cortes et al., 2022). There are also many attempts to applicate immunotherapy to glioma. However, almost all these attempts finally failed to improve overall survival (Weller et al., 2017; Wakabayashi et al., 2018; Reardon et al., 2020; Lim et al., 2022; Omuro et al., 2022). Many reasons were put forward to explain these failures. One important reason was that the blood-brain barrier (BBB) would prevent most peripheral immune cells, including circulating monocytes, naïve lymphocyte, and dendritic cells, from entering CNS and creating an immunologically quiescent microenvironment in CNS (Jackson et al., 2014; Jackson et al., 2019). However, an astonishing study revealed a novel lymphatic pathway for the egression of antigen-presenting cells from the brain (Louveau et al., 2015). Then the B and T lymphocytes outside the brain would be primed and deliver robust immune responses (Lim et al., 2018). These studies supported that the CNS was of distinct immune patterns, but if we could further elucidate and utilize these distinct features, there were also adequate opportunities to apply immunotherapy in glioma.

The reshaped metabolic patterns of the tumor have been noted for their interaction with immune responses (Xia et al., 2021). Purine metabolism, which could maintain cellular pools of guanylate and adenylate, was a critical compartment of cellular metabolism and a potential therapeutic target in cancers (Yin et al., 2018). Recent evidence also demonstrates that purinosomes, the punctate bodies in the cellular cytoplasm that activates the *de novo* purine biosynthesis, could interact with mitochondria, and regulate cell cycle (Yin et al., 2018). Purine metabolism has been proven to regulate the maintenance of glioma, initiating cells and apoptosis of astrocytes (Di Iorio et al., 2002; Wang et al., 2017). Furthermore, a recent study revealed that manipulation of purine metabolism could enhance the response to immune checkpoint inhibitors (Keshet et al., 2020). Besides, purine metabolic checkpoint could regulate autoimmunity (Saveljeva et al., 2022). Therefore, to explore if purine metabolism was involved in the pathophysiology of gliomas and the immune features of the glioma microenvironment, we analyzed the expression pattern of purine-metabolism-related genes (PuMG) in gliomas and investigated the correlation of purine-metabolism-related genes risk signature (PuMRS) with the clinicopathological characteristics, molecular features, and immunological landscapes of gliomas using public and in-house datasets.

Based on the different expression patterns of PuMGs, we first cluster the glioma patients into two subgroups. Distinctive patterns of clinicopathological features and prognosis of these two subgroups were introduced. Moreover, the functional gene sets enrichment analysis indicated that different expression patterns of PuMGs could regulate several pathways, including DNA replication and angiogenesis, which corroborated with the functions of purine metabolism. The activity of DNA replication was directly related to cell proliferation. Purine metabolism regulated the activity of DNA replication, suggesting the relationship between purine metabolism and tumor proliferation, which might lead to totally different prognosis in gliomas with different purine metabolism pattern. Besides, the response to IFN-γ was also significantly different in different clusters. IFN-γ was an important cause of PD-L1 expression and consequent immunosuppressive effect in glioma (Qian et al., 2018), suggesting the potential correlation between purine metabolism and immunosuppression, which might cause the poorer prognosis of cluster 2. The patterns of gene alterations were also different in these two subgroups. IDH mutation, recognized as an important marker for the classification and prognosis of glioma, would lead to production of the oncometabolite D-2- hydroxyglutarate and might reprogram the metabolism (Yan et al., 2009; Pirozzi and Yan, 2021). Additionally, codeletion of 1p/19q and IDH mutation with either mutant TERT promoter or ATRX has been reported to be closely associated with better prognosis of glioma patients (Eckel-Passow et al., 2015; Louis et al., 2021), and MGMT promoter methylation has been shown capable of predicting better response to temozolomide, a first-line chemotherapy for glioblastomas (Hegi et al., 2005). Our results revealed that the incidence of IDH mutation differs enormously in two subgroups, suggesting that the expression of purine metabolism-related genes might interact with IDH mutation in gliomas. However, even enrolling PuMRS with IDH mutation into multivariate analysis, the PuMRS was still proved as independent prognostic factor, indicating that PuMRS was a robust prognostic factor even after considering the correlation with IDH mutation. Furthermore, the alteration incidence of epidermal growth factor receptor (EGFR), which was recognized as essential for glioma development and frequently mutated, amplified, and overexpressed in malignant glioma (Eskilsson et al., 2018), was significantly different in two subgroups, suggesting potential interactions between purine metabolism and EGFR. Many EGFR-targeted therapies have been developed and evinced favorable efficacy in many tumors (Mok et al., 2009; Ramalingam et al., 2020). However, all attempts to improve the overall survival of glioma patients using EGFR inhibitors eventually failed (Chinot et al., 2014; Gilbert et al., 2014; Weller et al., 2017). Although the relationship between purine metabolism and EGFR remained unclear, our study found clues and might provide a novel direction for applications of EGFR-targeted therapies in glioma and overturn previous failures.

After filtering PuMGs, 11 PuMGs were recognized as essential genes for glioma prognosis. For example, Ribonucleotide Reductase Regulatory Subunit M2 (RRM2) was a catalytic subunit of ribonucleotide reductase and functioned as a critical enzyme in the process of DNA replication and repair (Chabes and Thelander, 2000; Torrents et al., 2002; Nordlund and Reichard, 2006), and RRM2 has been proved negatively correlated with prognosis of glioma (Sun et al., 2019). Furthermore, RRM2 could also facilitate immune infiltration of tumors (Tang et al., 2021). Our results revealed that RRM2 was a hazardous prognostic factor for glioma, and the subgroup with a higher expression level of RRM2 harbored more immune infiltration and expressed more immunotherapy-related markers, agreeing with the conclusions of previous studies. Besides, phosphodiesterase 2A (PDE2A) could regulate cyclic nucleotide signaling and the response to multiple stimulations (Barbagallo et al., 2020). Our results demonstrated that a higher expression level of PDE2A was correlated with better overall survival and less immune infiltration in glioma. The purine-metabolism-related genes signature (PuMRS) was constructed based on these 11 essential genes. It was manifested with strong potential to serve as a critical factor for prognosis prediction in glioma patients, indicating that PuMRS were tightly related to the prognosis of glioma.

Analyses of immune features depicted the correlation between purine metabolism and the immune microenvironment of glioma. The CIBERSORTx analysis determined higher infiltrations of several immune cells. For instance, M2 macrophage, which functioned as a critical role in tumor promotion and immunosuppressing (Noy and Pollard, 2014), was significantly more infiltrated into gliomas of the PuMRS high-risk group. Circulating monocytes and adjacent resident macrophages could be recruited to the tumor microenvironment and be polarized from M1 to M2 macrophages, forming tumor-associated macrophages (TAMs) (Anderson et al., 2021). TAMs could function to produce cytokines to suppress functions of T cells, secret chemokines to recruit Treg cells, and upregulate immunosuppressive surface proteins (Curiel et al., 2004; Colombo and Piconese, 2007; Yang and Zhang, 2017). These functions of TAMs contribute to immune escape in glioma, leading to a worse prognosis for high-risk group, which harbored more infiltration of TAMs. Moreover, TAMs could also express PD-L1 to inhibit tumor immunity and phagocytosis (Gordon et al., 2017), an essential target immune checkpoint inhibitor to enhance anti-tumor immunity (Cha et al., 2019). The expression level of PD-L1 was also remarkably higher in PuMRS high-risk group, suggesting that the higher expression level of immune checkpoints was related to poorer prognosis in glioma, which is consistent previous pan-cancer studies (Liu et al., 2020). Furthermore, the immunological “hot tumorˮ features were confirmed in the PuMRS high-risk group gliomas. These findings endorsed the potential ability of PuMRS to predict the immune characteristics of the tumor microenvironment in glioma. Those gliomas with high PuMRS would have more immune cell infiltration and overexpress several immunotherapy targets (PD-1, PD-L1, B7-H3, and CTLA4). Even though the overexpression of these markers might be correlated with a worse prognosis, a better response to immune checkpoint inhibitors might be accompanied by the overexpression of these immune-related markers. Consequently, PuMRS would help determine the immune characteristics and choose an optimal strategy for immunotherapy in gliomas.

Our present study investigated the relationship of PuMGs with the clinicopathological features and immunological characteristics of glioma. However, there are still some limitations to our study. First, the sequencing protocols and data preprocessing procedures differ among the four independent datasets. Second, some critical markers, including IDH mutation status, were unavailable in the REMBRANDT database. Besides, since the results of the current study were mostly derived from bulk RNA-sequencing data, we were not able to delineate the expression of PuMGs for each cell type in tumors. For the same reason, the findings about the relationship between purine metabolism-related genes and immune profiles were concluded based on analyses of mRNA expression rather than protein studies. Therefore, the findings of our study require future experimental validation to further elucidate the mechanism for the correlations, as well as the regulation of PuMGs and their downstream factors.

## Conclusion

In conclusion, we demonstrated that the expression of PuMGs was closely related to clinicopathological features and immune landscapes of glioma based on the comprehensive analyses of four independent datasets. The novel PuMRS showed up strong potential in predicting the prognosis of glioma patients. Moreover, it could also function as a potential marker for predicting immune cell infiltration and expression level of immunotherapy targets in gliomas. Based on these findings, we believe the PuMRS might be useful in directing immunotherapy in gliomas. Furthermore, we identified several essential PuMGs that influenced prognosis a lot, which might be a great resource for glioma studies to investigate the mechanisms of purine metabolism regulation and potential therapeutic targets.

## Data Availability

The datasets presented in this study can be found in online repositories. The names of the repository/repositories and accession number(s) can be found in the article/Supplementary Material.

## Supplementary material

SUPPLEMENTARY FIGURE S1
**(A)** Distribution of maximum FPKM values of genes in the TCGA dataset between 0–100. **(B)** Distribution of maximum FPKM values of genes in the TCGA dataset between 0–1. **(C)** Differences in the expression of 136 PuMGs between two consensus clusters. **P* < 0.05; ***P* < 0.01; ****P* < 0.001; *****P* < 0.0001.

SUPPLEMENTARY FIGURE S2 Differences in the clinicopathological features between two consensus clusters. **P* < 0.05; ***P* < 0.01; ****P* < 0.001; *****P* < 0.0001.

SUPPLEMENTARY FIGURE S3 Representative immunohistochemical staining for POLR1D and PDE2A from the Human Protein Atlas. **(A)** Representative IHC staining for POLR1D in high- and low-grade gliomas (https://www.proteinatlas.org/ENSG00000186184-POLR1D/, antibody: HPA039337). **(B)** Representative IHC staining for PDE2A in high- and low-grade gliomas (https://www.proteinatlas.org/ENSG00000186642-PDE2A/, antibody: CAB009752).

SUPPLEMENTARY FIGURE 4 Differences in the clinicopathological features between two PuMRS risk groups. **P* < 0.05; ***P* < 0.01; ****P* < 0.001; *****P* < 0.0001.

SUPPLEMENTARY FIGURE 5 Differences in immunological characteristics of tumor microenvironment between two PuMRS risk groups in CGGA, REMBRANDT, and WCH cohorts. **P* < 0.05; ***P* < 0.01; ****P* < 0.001; *****P* < 0.0001.
