# Supplementary figures and images for: Purine metabolism-related gene expression signature predicts survival outcome and indicates immune microenvironment profile of gliomas
Source: Front Pharmacol. 2022 Nov 10;13:1038272. doi: 10.3389/fphar.2022.1038272 (PMC9685320; doi:10.3389/fphar.2022.1038272)

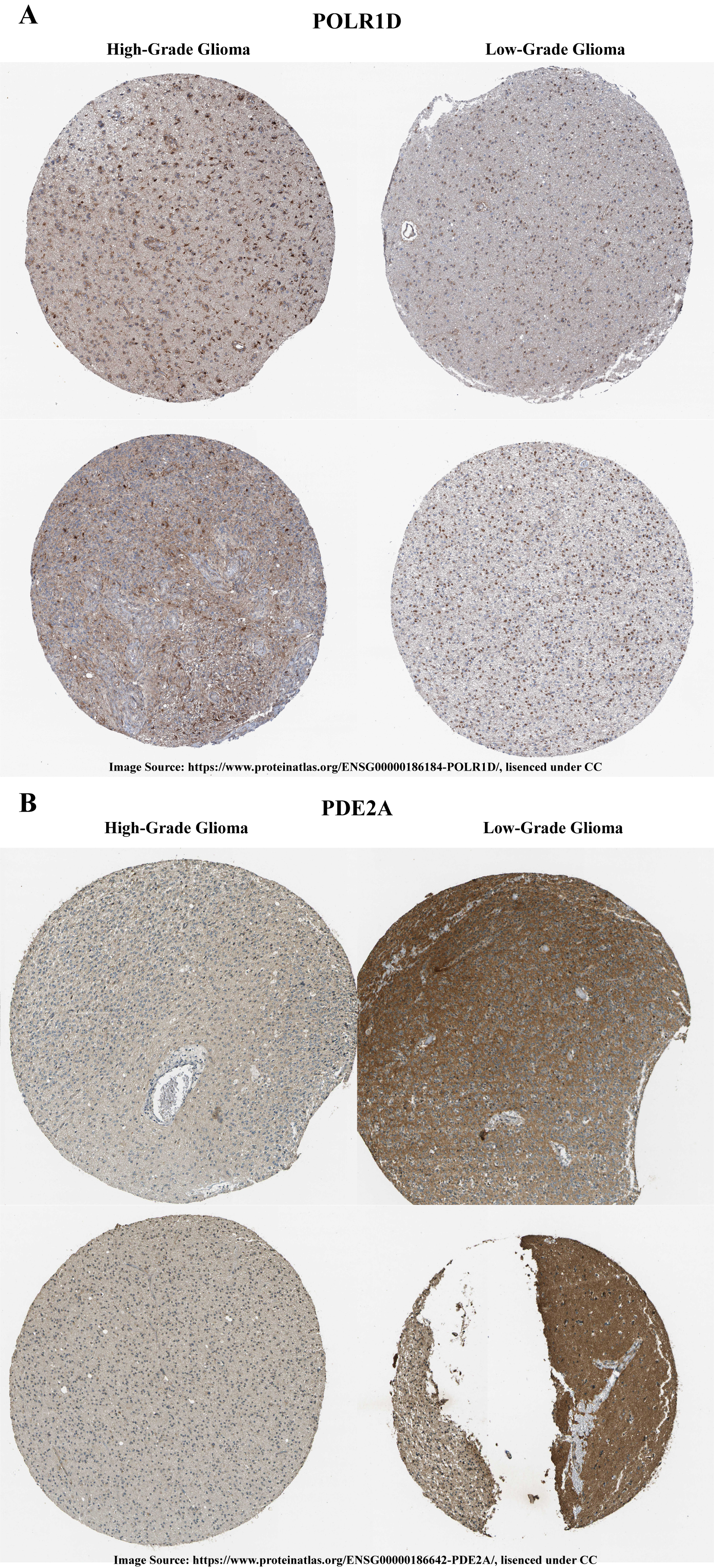

Supplement: Supplementary file 2 [file Image3.TIF]

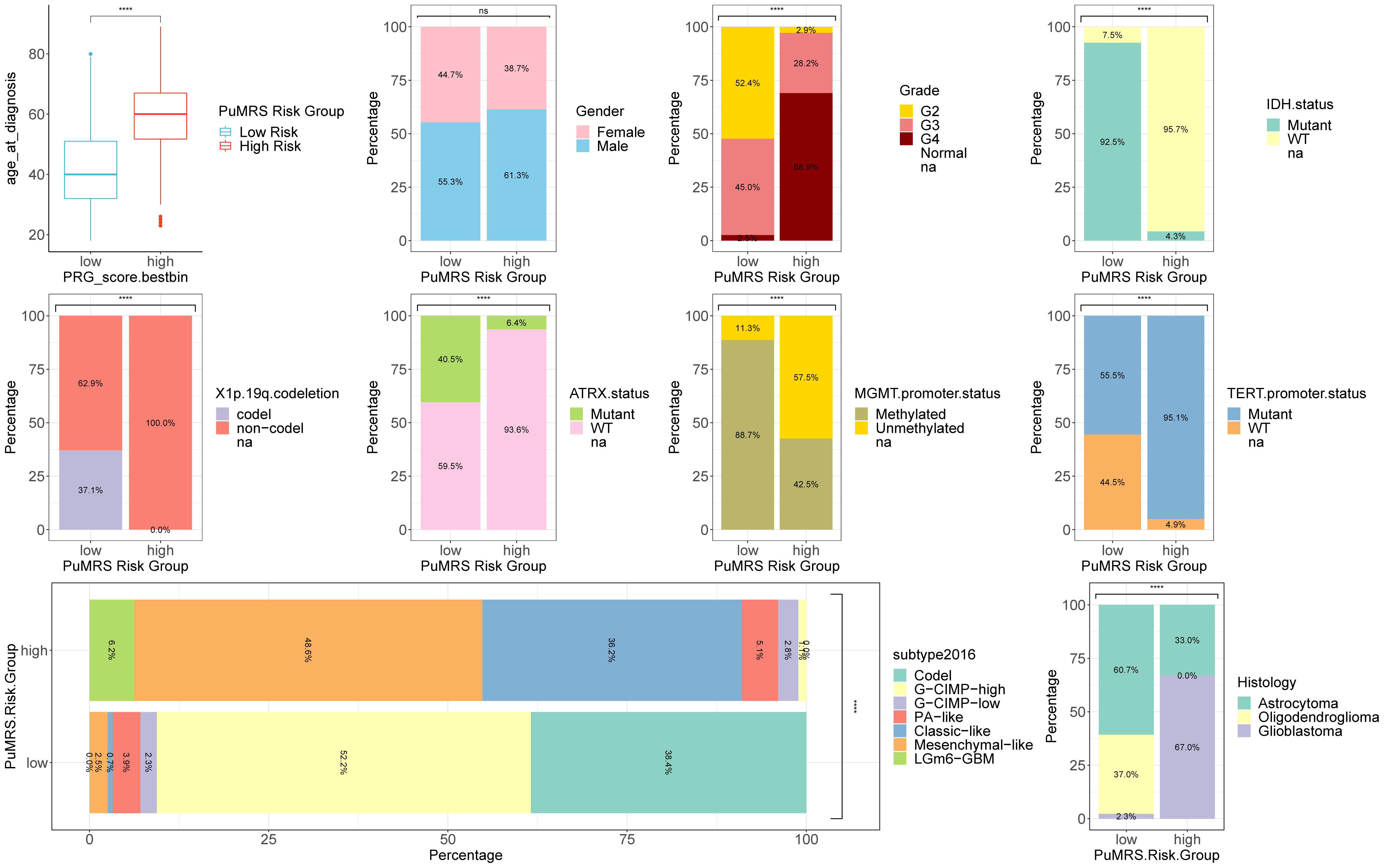

Supplement: Supplementary file 4 [file Image4.TIF]

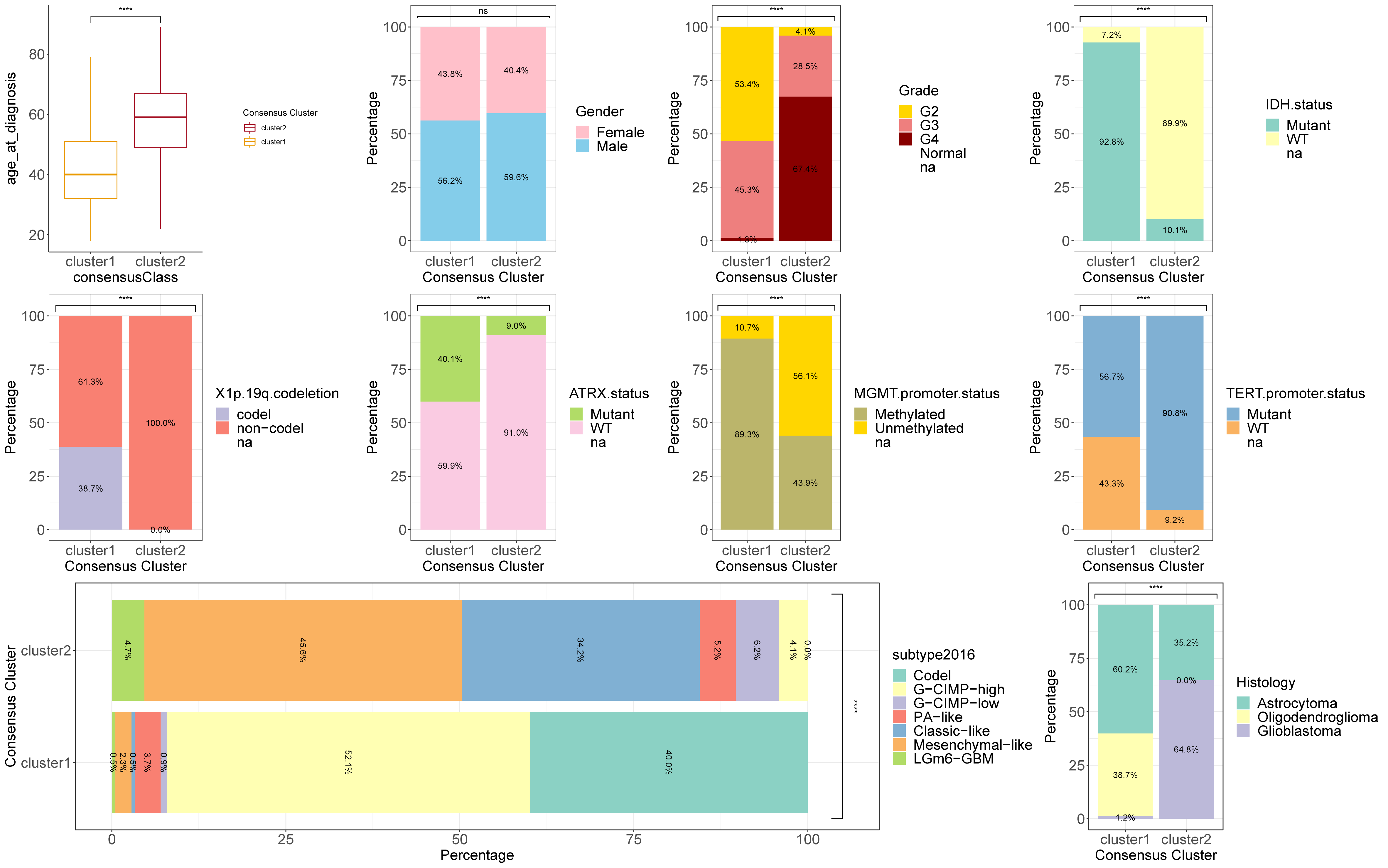

Supplement: Supplementary file 5 [file Image2.TIF]

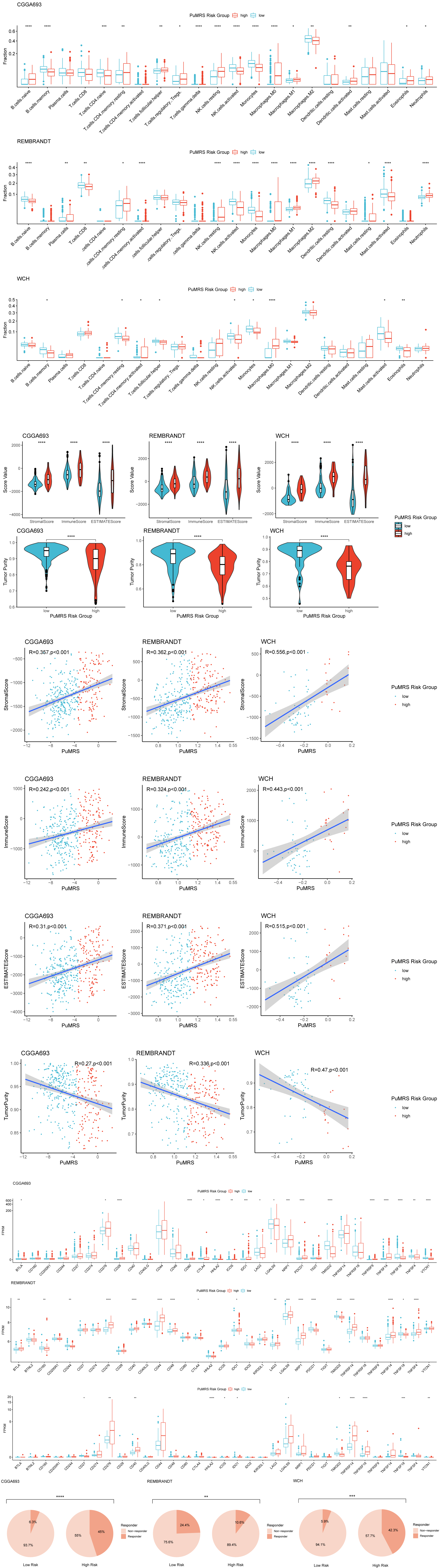

Supplement: Supplementary file 9 [file Image5.TIF]
